# Supplementary material for: Content-rich biological network constructed by mining PubMed abstracts
Source: BMC Bioinformatics. 2004 Oct 8;5:147. doi: 10.1186/1471-2105-5-147 (PMC528731; doi:10.1186/1471-2105-5-147)
Supplement: Additional File 5 — The original Chilibot query results of the term "long-term potentiation (LTP)" and 22 other terms, limiting the latest references analyzed to the years 1990, 1995, 2000, and 2004. [file 1471-2105-5-147-S5.bz2 › chilibotAdditionalFile5/ltp2000/html/PKA.html]

 


**PKA** (Input: PKA ) 

---


**Google Searches:** Entire Web  | EDU domain only  | PDF files only

.

**External Links:** OMIM |
LocusLink
| Swissprot
| GeneCards

  
**Maps of PKA**

Simple
Complete
graph in
radiant
tree
square
layout.

**New Hypothesis !**

**Synonyms** 

- pka   [PubMed]

**Synopsis**

- These data demonstrate a role of **PKA** in the induction and NOT the expression of cortical LTP and suggest that if **PKA** is involved in the late phase of LTP, it does NOT appear to be a persistent activation.  Eur J Neurosci, 1998 
  [26]

- We suggest that neither PKC nor **PKA** is required to induce LTP at this synapse.  Eur J Neurosci, 2000 
  [25]

- These results suggest that **PKA** and MAPK are critical for the expression of a persistent phase of LTP in the lateral amygdala.  J Neurosci, 2000 
  [23]

- These results show that induction of cerebellar LTP requires a presynaptic signaling cascade, including Ca influx, stimulation of Ca sensitive adenylyl cyclase, and activation of **PKA**, and argue against a requirement for postsynaptic Ca signals or their sequelae.  J Neurosci, 1999 
  [20]

- These results suggest that NO contributes to L LTP by stimulating guanylyl cyclase and cGMP dependent protein kinase, which acts in parallel with **PKA** to increase phosphorylation of the transcription factor CREB.  J Neurosci, 1999 
  [20]

- These results are consistent with the hypothesis that the developmental changes in phosphorylation of endogenous substrates by **PKA** is controlled largely by changes in the concentration of those substrates.  Neurochem Res, 1991 
  [20]

- Thus, we further show that, in both PC12 cells and hippocampal neurons, depolarization induced calcium influx stimulates ERK activity in a **PKA** dependent manner.  J Biol Chem, 2000 
  [19]

- Our results show that, like non theta LTP, theta induced LTP in area CA1 of the mouse hippocampus involves transcription, translation, and **PKA** and suggest that cAMP mediated gene transcription may be a common mechanism responsible for the late phases of LTP induced by both theta and non theta patterns of stimulation.  Learn MemLearn Mem, 2000 
  [17]

- Thus simultaneous presynaptic generation of cyclic GMP and inhibition of **PKA** is sufficient to induce LTD of synaptic transmission at Schaffer collateral CA1 synapses.  J Neurophysiol, 1999 
  [17]

- The similar time courses of synaptic, network, and behavioral instability suggest that the genetic reduction of **PKA** activity is responsible for the defects at each level and support the idea that hippocampal synaptic plasticity is important in spatial memory.  J Neurosci, 2000 
  [16]

- It is suggested that the induction of LTD by LFS involves activation of PKC and **PKA** following activation of group I and group II metabotropic glutamate receptors mGluR .  Neurosci Lett, 1999 
  [16]

- . . . the increase in CREB phosphorylation was NOT, which suggests that in area CA3 the **PKA** cascade is NOT coupled to CREB phosphorylation via ERK.  J Neurosci, 2000 
  [15]

- Our results support the idea of a postsynaptic mechanism maintaining late phase LTP, in which AMPA receptor synthesis is increased as a result of **PKA** dependent gene transcription.  Nature, 1998 
  [14]

- Both activation and inhibition of GABA A receptor currents as a result of **PKA** and PKC phosphorylation have been reported, while phosphorylation by PTK enhances function.  Adv Second Messenger Phosphoprotein Res, 1999 
  [14]

- **PKA** activation is observed in the induction of LTP.  Biochem Biophys Res Commun, 2000 
  [13]
